# 

**Published:** 1808-01

**Authors:** 


					" J 7 k / 7 <(!>
THE - -i/
>"
MEDICAL
AND
PHYSICAL
JO U M JV A L,
CONDUCTED BY
T. BRADLEY, M. D.
AND
R.* BATTY, M.D.
VOL. XIX.
From January to June, 1808.
Ay 2 S
LONDON:
PRINTED FOR RICHARD PHILLIPS, NO. .6, BRIDGE-STREET.
BLACKFRIARS.
By William Thornc, ReJ Lion Courts Fleet Sues'-
: - 1 W
... 1-
HE 18 Z
L CnreceU at fetatfoncctf ^all. ]

				

